# Highly Pathogenic Avian Influenza A(H5N8) Viruses Reintroduced into South Korea by Migratory Waterfowl, 2014–2015

**DOI:** 10.3201/eid2203.151006

**Published:** 2016-03

**Authors:** Jung-Hoon Kwon, Dong-Hun Lee, David E. Swayne, Jin-Yong Noh, Seong-Su Yuk, Tseren-Ochir Erdene-Ochir, Woo-Tack Hong, Jei-Hyun Jeong, Sol Jeong, Gyeong-Bin Gwon, Chang-Seon Song

**Affiliations:** Konkuk University, Seoul, South Korea (J.-H. Kwon, J.-Y. Noh, S.-S. Yuk, T.-O. Erdene-Ochir, W.-T. Hong, J.-H. Jeong, S. Jeong, G.-B. Gwon, C.-S. Song);; S Department of Agriculture, Athens, Georgia, USA (D.H. Lee, D.E. Swayne)

**Keywords:** highly pathogenic avian influenza virus, H5N8, South Korea, wild waterfowl, phylogenetic analysis, influenza, viruses

## Abstract

Highly pathogenic avian influenza A(H5N8) viruses were isolated from migratory waterfowl in South Korea during fall 2014–winter 2015, a recurrence after initial introduction in winter 2014. These reappeared viruses were phylogenetically distinct from isolates circulating in poultry farms in South Korea.

Since the Asian-lineage subtype H5 highly pathogenic avian influenza (HPAI) virus was first detected in China in 1996, outbreaks of infection caused by this virus in poultry have been continuous. The HPAI (H5) viruses have evolved and continue to evolve into many genetic lineages and multiple clades (*1*). In January 2014, novel reassortant HPAI viruses of subtype H5N8, clade 2.3.4.4, were detected in poultry and wild bird carcasses in South Korea (*2*). Closely related viruses were also detected in Japan (*3*) and China (*4*). Genetic analysis showed that this virus was generated by reassortment of HPAI viruses of eastern China. Subsequently, HPAI (H5N8) viruses spread to Europe and North America and were then reintroduced into South Korea and Japan (*5*). The HPAI (H5N8) viruses identified in South Korea in early 2014 were divided into groups A (A/Baikal teal/Korea/Donglim3/2014 strain-like) and B (A/breeder duck/Korea/Gochang1/2014-like). Group A viruses further evolved into 3 distinct subgroups: icA1 (Europe/Japan), icA2 (North America/Japan), and icA3 (South Korea/Japan) (*5*). Wild birds were suspected of being a source of intercontinental transmission because the timing and direction of the outbreak coincided with the migratory route of wild birds (*5*,*6*). We sequenced and genetically analyzed the complete genomes of 11 HPAI (H5N8) viruses isolated from wild migratory waterfowl in South Korea during December 2014 and February 2015 and compared these isolates with other HPAI (H5N8) isolates, including isolates identified from South Korea poultry farms in late 2014.

## The Study

A total of 11 HPAI (H5N8) viruses were isolated from 980 samples of wild bird feces and 102 swab samples collected from wild bird habitats in South Korea where active surveillance was conducted during December 2014 and February 2015 (Table). Eight of 65 fecal samples (K14-362–K14-374) collected on December 2014, one of the 50 fecal samples (N15-99) collected on February 2015, one of the 17 swab samples from healthy common teals (KU-12) collected on January 2015, and one of the 13 swab samples from healthy mallards (KU3-2) collected on February 2015 were positive for influenza A virus by egg inoculation and matrix (M) gene real-time reverse transcription PCR performed as described (*8*). The hosts of the influenza A virus–positive fecal samples were identified as mandarin ducks, greater white-fronted geese, and mallards by DNA barcoding techniques, as described (*7*). Full-genome sequencing was performed by next-generation sequencing using the Ion Torrent Personal Genome Machine system (Thermo Fisher Scientific, Grand Island, NY, USA) (Technical Appendix 1). The viruses were subtyped as H5N8 by using a BLAST search, and the multibasic cleavage site of the hemagglutinin (HA) gene (PLRERRRKR/GLF) was detected.

**Table:** HPAI (H5N8) isolates and total wild bird samples collected in South Korea, December 2014–February 2015*

Collection date	Location	Sample type	No. HPAI (H5N8) positive/no. total	Host†	Strain
2014					
Sep 26	36°44′N, 127°07′E	Feces	0/55		
Sep 27	36°37′N, 126°21′E	Feces	0/110		
Nov 6	36°37′N, 126°21′E	Feces	0/335		(1 LPAI)
Nov 7	36°44′N, 127°07′E	Feces	0/105		
Nov 22	36°44′N, 127°07′E	Feces	0/260		(3 LPAI)
Dec 24	36°44′N, 127°07′E	Feces	**8/65**	Mandarin duck	K14-363-1
					K14-366-1
					K14-367-1
				Greater white-fronted goose	K14-367-4
					K14-369-3
					K14-371-4
					K14-372-2
					K14-374-1
2015					
Jan 22	36°47′N, 127°03′E	Swab	**1/17**	Common teal	KU-12
Jan 29	35°18′N, 128°40′E	Swab	**1/13**	Mallard	KU3-2
			0/30	Northern pintail	(1 LPAI)
Feb 6	37°32′N, 127°01′E	Feces	1/50	Mallard	N15-99
Feb 11	36°42′N, 126°27′E	Swab	0/14	Mallard	
Feb 25	37°23′N, 129°14′E	Swab	0/13	Black-tailed gull	
Mar 16	35°53′N, 127°01′E	Swab	0/15	Eurasian wigeon	
Total			11/1,082		

*Boldface indicates period of study. HPAI, highly pathogenic influenza virus; LPAI, low pathogenicity avian influenza. †The hosts of the HPAI-positive fecal samples were identified by using DNA barcoding techniques as described (*7*).

For phylogenetic analysis, we constructed a maximum-likelihood tree in MEGA6 software (http://www.megasoftware.net) using the Hasegawa-Kishino-Yano (HKY) model. A median-joining phylogenetic network was constructed by using NETWORK version 4.613 (www.fluxus-engineering.com), and Bayesian analysis was performed by using BEAST version 1.8.1 (http://beast.bio.ed.ac.uk). A maximum clade credibility tree was generated for each dataset by using TreeAnnotator in BEAST (Technical Appendix 1).

**Figure 1:**
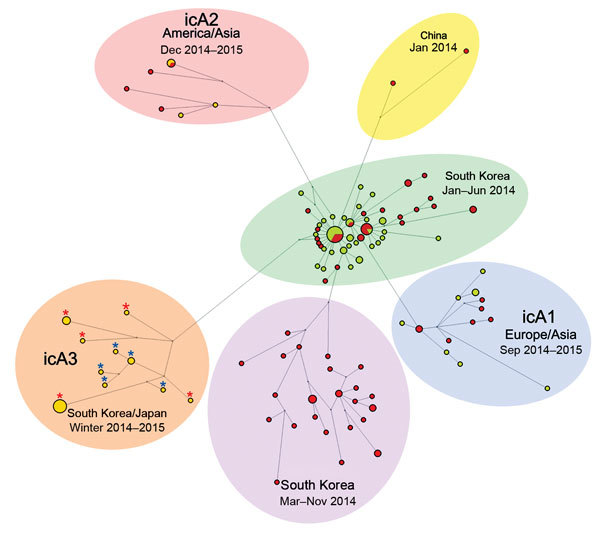
Median-joining phylogenetic network of highly pathogenic avian influenza A(H5N8) viruse isolates identified in South Korea during 2014–2015 showing relationships with other virus isolates. The median-joining network was constructed from the hemagglutinin gene and includes all the most parsimonious trees linking the sequences. Each unique sequence is represented by a circle sized relative to its frequency in the dataset. Branch length is proportional to the number of mutations. Isolates are colored according to the origin of the sample: red inner circle, poultry farm isolates; yellow inner circle, wild bird isolates. Red asterisks indicate isolates from South Korea and blue asterisks indicate isolates from Japan identified during December 2014–February 2015.

**Figure 2:**
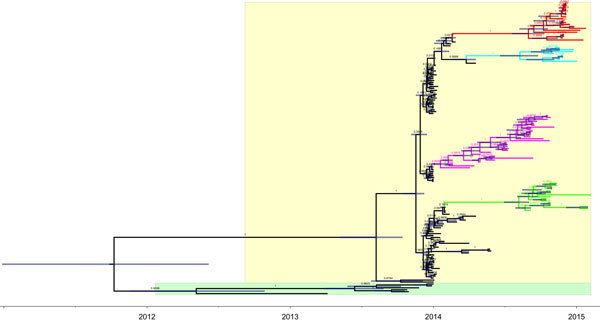
Temporally structured maximum clade credibility phylogenetic tree (years shown on the horizontal axis) of the hemagglutinin gene of highly pathogenic avian influenza (H5 clade 2.3.4.4) viruses. Yellow, group A; green, group B. Branches of group A are colored according to the origin of the sample: purple, isolates from poultry farms (South Korea); blue-green, icA2 (North America, Japan); green, icA1 (Europe, South Korea, Japan); red, icA3 (South Korea, Japan).

Each genome segment of 11 HPAI (H5N8) viruses shared high nucleotide sequence identities ranging from 99.1% to 100%: polymerase basic protein 2, 99.4%–100%; polymerase basic protein 1, 99.3%–100%; polymerase acidic protein, 99.5%–100%; HA, 99.1%–100%; nucleoprotein, 99.6%–100%; neuraminidase, 99.2%–100%; M protein, 99.4%–100%; and nonstructural protein, 99.2%–100%. Phylogenetic analysis showed that the 4 different subtype H5N8 virus clusters, icA 1–3 and the South Korea poultry farm cluster, most likely evolved from H5N8 virus identified from South Korea in early 2014. All H5N8 isolates collected in South Korea during winter 2014–15 identified in this study clustered with isolates from Japan, including the A/chicken/Miyazaki/7/2014 strain, and were characterized as subgroup icA3. Isolates obtained from South Korea poultry farms in late 2014 were phylogenetically distinct from isolates in other subgroups (Figure 1; Technical Appendix 1 Figures 1, 2).

Group A (H5N8) viruses have been detected on South Korea poultry farms since the first outbreak in January 2014, including during the summer season. A second wave of the HPAI (H5N8) outbreak started in September 2014. Although the growing HPAI outbreak in September 2014 coincided with the fall migration of migratory waterfowl, phylogenetic analyses suggest that the HPAI (H5N8) viruses detected on South Korean poultry farms in late 2014 are not related to the icA3 viruses carried by wild waterfowl but have instead evolved from viruses circulating on poultry farms or among resident wild birds in South Korea since early 2014.

By the beginning of the fall 2014 migration of migratory waterfowl, new subgroups of H5N8 viruses (icA1, icA2, icA3) were detected in wintering sites of migratory waterfowl, including South Korea and Japan, in late 2014 and early 2015 (*5*,*9*). The icA1 subgroup is composed of HPAI (H5N8) viruses from Europe, South Korea, and Japan, whereas the icA2 subgroup is composed of HPAI (H5N8) viruses from North America, Taiwan, and Japan and the icA3 subgroup is composed of HPAI (H5N8) viruses isolated in South Korea and Japan. Markov chain Monte Carlo analyses showed that the substitution rates estimated for HPAI (H5N8) viruses identified from South Korea are 9.23 × 10^−3^ (95% highest posterior density range 7.43 × 10^−3^ to 1.11 × 10^−2^) nt substitutions/site/year, which is higher than previous estimates for the HA gene of H5N1 viruses from China from 1996 through 2012 (4.378 × 10^−3^ nt substitutions/site/year] (*10*). The interval estimated from most recent common ancestor of the icA3 cluster from South Korea and Japan was 0.44 years (95% highest posterior density range 0.33–0.55 months, corresponds to August 2014) (Figure 2). 

## Conclusions

These results suggest that HPAI (H5N8) viruses circulated in wild bird populations and evolved into subgroups during the breeding season. Detection of subtype H5N8 viruses in healthy wild birds (*12**,**13*; this study) and subclinical infection with viral shedding among migratory waterfowl experimentally infected with HPAI (H5N8) viruses (*11*) support the theory of long-term circulation of HPAI (H5N8) viruses in wild bird population.

This study also found that subtype icA3 viruses, derived from HPAI (H5N8) viruses from South Korea and reintroduced by migratory waterfowl, were genetically distinct from the HPAI (H5N8) viruses that continued to circulate in poultry farms. In the previous 4 HPAI (H5N8) virus outbreaks in South Korea and Japan, migratory waterfowl were identified as the source of HPAI outbreaks (*14**,**15*); however, related HPAI viruses were not reintroduced into South Korea and Japan after the initial outbreak season. The phylogenetic analysis described here shows that HPAI (H5N8) viruses isolated from migratory wild birds in the winter of 2014–15 are phylogenetically distinct from isolates from South Korean poultry farms. HPAI (H5N8) viruses thus independently evolved in wild bird populations and poultry farms in South Korea until late 2014.

Our results indicate that HPAI (H5N8) viruses have been circulating in wild waterfowl population since early 2014. Enhanced global active surveillance is needed to monitor the spread of these viruses through wild birds. Such efforts could clarify the epidemiology of HPAI virus and facilitate early recognition of novel genotypes.

Technnical Appendix 1 Methods for genetic characterization of highly pathogenic avian influenza (H5N8) viruses in South Korea, winter of 2014–2015.

Technical Appendix 2 Sequences from the Global Initiative on Sharing All Influenza Data EpiFlu Database on which this research is based.
