# Supplementary material for: Highly Pathogenic Avian Influenza A(H5N8) Viruses Reintroduced into South Korea by Migratory Waterfowl, 2014–2015
Source: Emerg Infect Dis. 2016 Mar;22(3):507–10. doi: 10.3201/eid2203.151006 (PMC4766904; doi:10.3201/eid2203.151006)
Supplement: Technnical Appendix 1 — Methods for genetic characterization of highly pathogenic avian influenza (H5N8) viruses in South Korea, winter of 2014–2015. [file 15-1006-Techapp-s1.pdf]

Article doi: http://dx.doi.org/10.3201/eid2203.151006

Technical Appendix 1

Methods for Genetic Characterization of Highly Pathogenic Avian Influenza, Subtype H5N8, Viruses in South Korea, Winter 2014–2015

Full-Genome Sequencing

For molecular analysis, RNA was extracted by using the RNeasy kit (QIAGEN, Valencia, CA, USA) according to the manufacturer's instructions. The 8 genes of each virus were amplified by using 2-step reverse transcription PCR. The reverse transcription PCR (RT-PCR) amplicons (2 μg) of all 8 gene segments was used to prepare Ion Fragment sequencing libraries (Life Technologies, Carlsbad, CA, USA) according to the manufacturer's instructions. Briefly, amplicons were loaded onto beads, and emulsion PCR was conducted before sequencing with an Ion 318 chip on an Ion Torrent Personal Genome Machine. De novo and directed assembly of genome sequences were performed by using Geneious R7 software (http://www.geneious.com).

Phylogenetic Analysis

For phylogenetic analysis, the nucleotide sequences used in this study were deposited in the database of the Global Initiative on Sharing All Influenza Data (www.gisaid.org) and in GenBank (www.ncbi.nlm.nih.gov/genomes/FLU). Complete coding regions were aligned by using MUSCLE, and manual editing and tree reconstruction were carried out using MEGA 6 (www.megasoftware.net). A maximum likelihood tree was estimated by the MEGA 6 software using the Hasegawa-Kishino-Yano model of nucleotide substitution with γ-distributed rate variation among sites with 4 rate categories. Statistical analysis of the phylogenetic tree was performed by bootstrap analysis carried out on 1,000 replicates. A median-joining phylogenetic network was constructed by using NETWORK ver. 4.613 (www.fluxus-engineering.com). Bayesian analysis performed with BEAST v1.7 (http://beast.bio.ed.ac.uk). A Markov chain Monte Carlo method was employed, and the associated evolutionary parameters were the codon-based SRD06 nt substitution model with uncorrelated lognormal relaxed clock and the Bayesian

skyline coalescent prior. The BEAST output was analyzed by using TRACER v1.4 in BEAST with 10% burn-in. All parameter estimates for each run showed effective sample size values >200. A maximum clade credibility tree was generated for each dataset by using TreeAnnotator in BEAST. FigTree 1.3.1 (http://tree.bio.ed.ac.uk/) was used for visualization of trees.

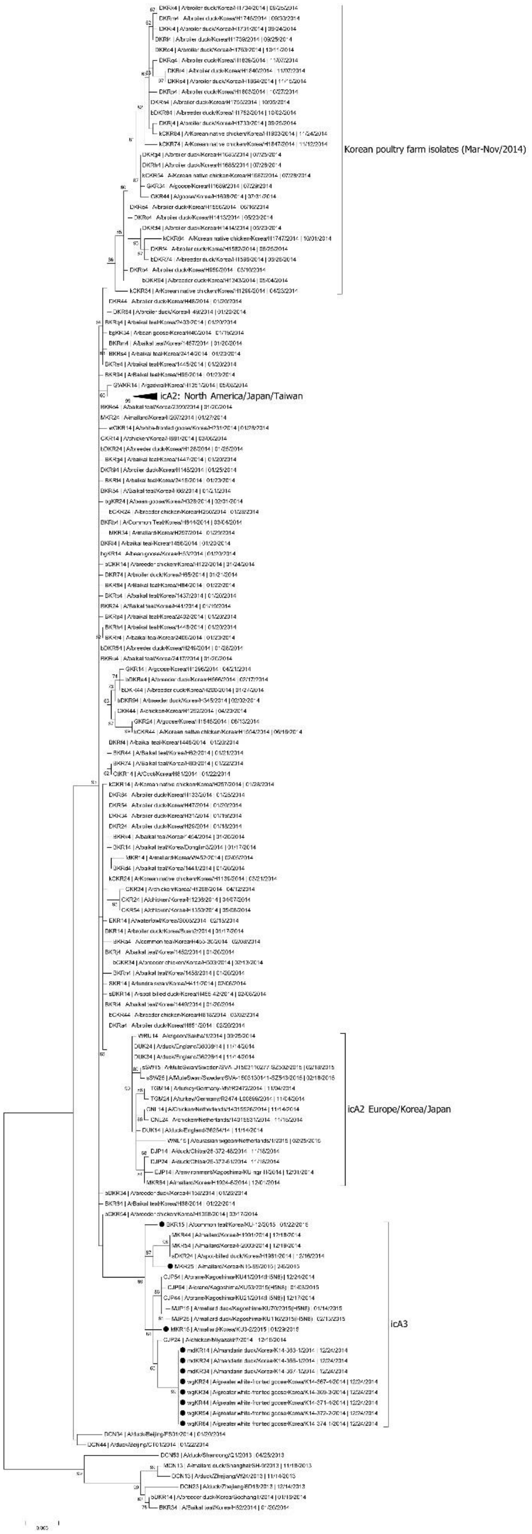

Technical Appendix Figure 1. Maximum likelihood phylogenetic tree of the hemagglutinin (HA) gene. The black circle identifies the HA gene of highly pathogenic avian influenza, subtype H5N8, isolates used in this study. The percentages of replicate trees in which the associated taxa clustered together in the bootstrap test (1,000 replicates) are shown next to the branches.

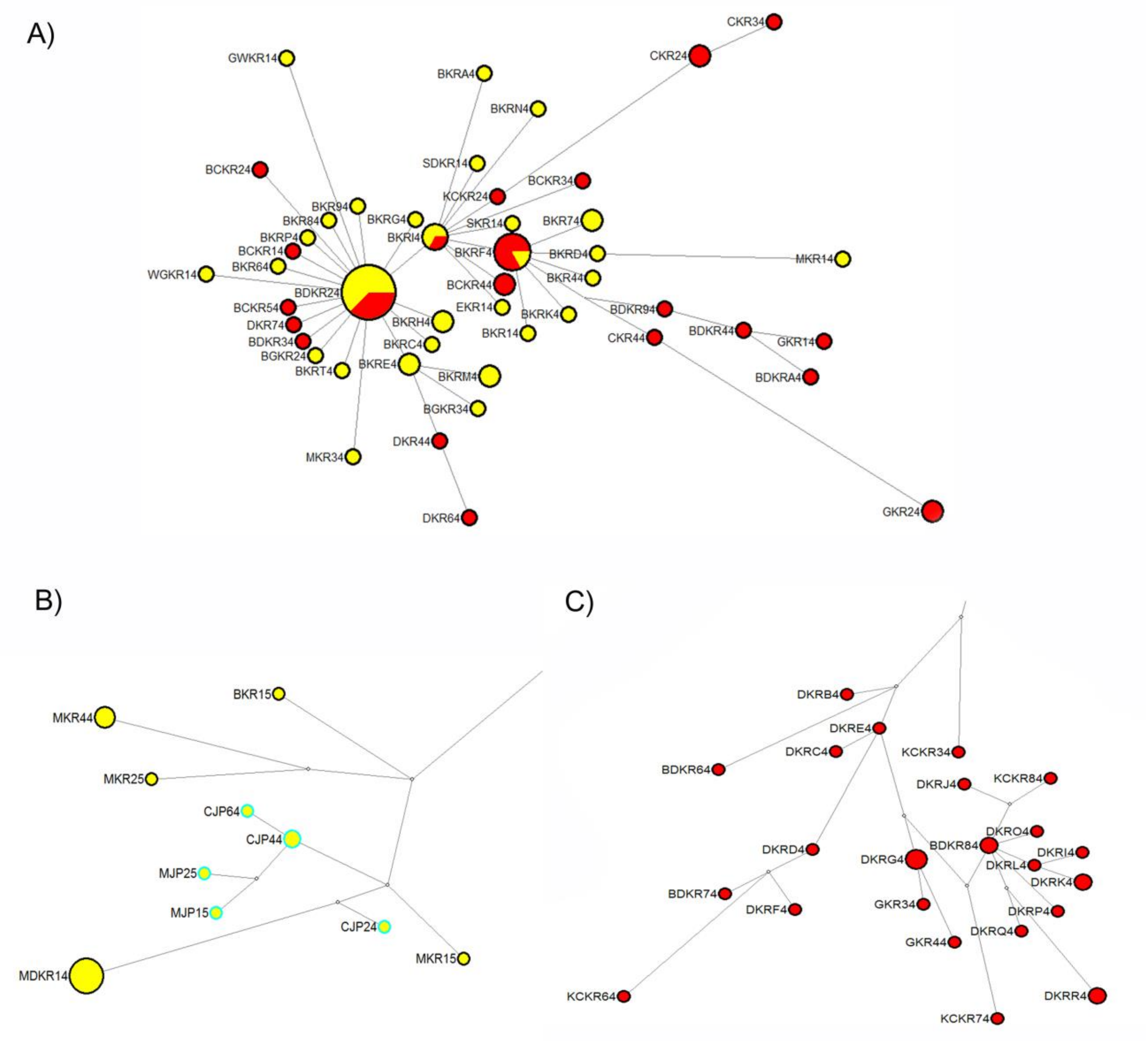

Technical Appendix Figure 2. Median-joining phylogenetic network of highly pathogenic avian influenza, subtype H5N8, viruses identified from South Korea in 2014–2015. A) Isolates obtained during January–June 2014. B) icA3. C) Isolates obtained during March–November 2014. The median-joining network was constructed from the hemagglutinin gene and includes all the most parsimonious trees linking the sequences. Each unique sequence is represented by a circle sized relative to its frequency in the dataset. Branch length is proportional to the number of mutations. Isolates are colored according to the origin of the sample: red inner circle, poultry farm isolates; yellow inner circle, wild bird isolates; black outer circle, isolates from South Korea; blue outer circle in B), isolates from Japan.
